# Pt(II) and Pt(IV) complexes with large hydrophobic ligands: a study of new potential cytostatics

**DOI:** 10.1186/1758-2946-5-S1-P52

**Published:** 2013-03-22

**Authors:** Petr Kacer, Jan Svoboda, Tereza Louzilova, Kamila Syslova, Marek Kuzma

**Affiliations:** 1 ICT - Prague, Technická 5, 166 28 Prague, Czech Republic; 2 Institute of Microbiology, Academy of Sciences, Vídeňská 1083, 142 20, Prague 4, Czech Republic

## 

Breast cancer is a major health problem among women in the world. The successful treatment of this disease is limited by the fact that essentially all breast cancers become resistant to chemotherapy. Therefore, there is a need to design new chemotherapeutic agents able not only to target breast cancer but also to display increased efficacy and overall decreased systemic toxicity. Platinum (II) complexes are widely used in cancer chemotherapy. The most important platinum-based drugs are cisplatin, carboplatin, the first platinum (II) derivatives entering the market, and more recently oxaliplatin, nedaplatin, iobaplatin, heptaplatin and picoplatin The computer-aided calculations are commonly applied to rational design of many pharmacological group of drugs. Platinum based cytostatics are one of the exceptions. The correlation between antiproliferative activity and molecular descriptors of Pt-drug analogues of clinically used complexes are present in this work. The main goal of this study was to show the relations between hydrophobicity and biological activity in the series of neutral platinum (II) and platinum(IV) complexes. The object of the study is several groups of analogues of oxaliplatin and picoplatin complexes, with N-donors ligands (amidines, adamantines and memantines). The influence of the type and the positions of the substituents on the conformational energies and thermodynamic stabilities of a series of platinum (II) and platinum (IV) complexes has been studied by molecular mechanics. The calculations were carried out for the ligand conformations. The obtained energies and thermodynamic stabilities are in agreement with experimental data on the reactivity and antitumor activity of the compounds.

